# Negative Epistasis between Sickle and Foetal Haemoglobin Suggests a Reduction in Protection against Malaria

**DOI:** 10.1371/journal.pone.0125929

**Published:** 2015-05-12

**Authors:** Bruno P. Mmbando, Josephine Mgaya, Sharon E. Cox, Siana N. Mtatiro, Deogratias Soka, Stella Rwezaula, Elineema Meda, Evarist Msaki, Robert W. Snow, Neal Jeffries, Nancy L. Geller, Julie Makani

**Affiliations:** 1 Muhimbili University of Health and Allied Sciences, Dar-es-Salaam, Tanzania; 2 National Institute for Medical Research, Tanga, Tanzania; 3 London School of Hygiene & Tropical Medicine, London, United Kingdom; 4 Muhimbili National Hospital, Dar-es-Salaam, Tanzania; 5 KEMRI-Wellcome Trust Programme, Nairobi, Kenya; 6 University of Oxford, Oxford, United Kingdom; 7 National Heart, Lung, and Blood Institute (NHLBI), National Institutes of Health (NIH), Bethesda, Maryland, United States of America; Sickle Cell Unit, JAMAICA

## Abstract

**Background:**

Haemoglobin variants, Sickle (HbS) and foetal (HbF) have been associated with malaria protection. This study explores epistatic interactions between HbS and HbF on malaria infection.

**Methods:**

The study was conducted between March 2004 and December 2013 within the sickle cell disease (SCD) programme at Muhimbili National Hospital, Tanzania. SCD status was categorized into HbAA, HbAS and HbSS using hemoglobin electrophoresis and High Performance Liquid Chromatography (HPLC). HbF levels were determined by HPLC. Malaria was diagnosed using rapid diagnostic test and/or blood film. Logistic regression and generalized estimating equations models were used to evaluate associations between SCD status, HbF and malaria.

**Findings:**

2,049 individuals with age range 0-70 years, HbAA 311(15.2%), HbAS 241(11.8%) and HbSS 1,497(73.1%) were analysed. At enrolment, malaria prevalence was significantly higher in HbAA 13.2% compared to HbAS 1.24% and HbSS 1.34% (p<0.001). Mean HbF was lower in those with malaria compared to those without malaria in HbAA (0.43% vs 0.82%) but was the reverse in HbSS (8.10% vs 5.59%). An increase in HbF was associated with a decrease in risk of malaria OR=0.50 (95%CI: 0.28, 0.90; p=0.021) in HbAA, whereas for HbSS the risk of malaria increased OR=2.94 (1.44, 5.98; p=0.003). A similar pattern was seen during multiple visits; HbAA OR=0.52 (0.34, 0.80; p=0.003) vs HbSS OR=2.01 (1.27, 3.23; p=0.003).

**Conclusion:**

Higher prevalence of malaria in HbAA compared to HbAS and HbSS confirmed the protective effect of HbS. Lower prevalence of malaria in HbAA with high HbF supports a protective effect of HbF. However, in HbSS, the higher prevalence of malaria with high levels of HbF suggests loss of malaria protection. This is the first epidemiological study to suggest a negative epistasis between HbF and HbS on malaria.

## Background

Sickle (HbS) and fetal hemoglobin (HbF) are hemoglobin variants that confer immunity against malaria [1–4]. HbS is a pathological haemoglobin variant resulting from the mutation on the β-globin gene on chromosome 11. It is inherited in Mendelian manner, with protection against malaria in both the heterozygous state HbAS [1,2] and the homozygous state HbSS [3]. HbF is a normal haemoglobin variant which is the predominant hemoglobin during the fetal period. The level of HbF is 100% at birth, but decreases in the first five years of life to reach a level of less than 2% in normal (HbAA) individuals [5]. In individuals with HbSS, the level of HbF is variable but may reach 20%, with high levels of HbF associated with amelioration of disease severity [7]. Although the relationship between HbF and malaria has been less well described, HbF is also associated with reduced risk of malaria [4,8]. The malaria hypothesis proposes that areas with high malaria transmission will have a high prevalence of malaria-protective haemoglobin variants [9]. However, epistatic interaction between genes has been described with malaria-protective genes, when the presence of one gene influences the phenotypic expression of another gene. Negative epistasis has been reported between sickle hemoglobin and α-thalassaemia, where their co-existence results in the loss of the malaria protection effect of either Hb variant [10]. In this study, we set out to explore the epistatic interaction between HbS and HbF on malaria in Tanzania, a country with a high prevalence of malaria and SCD.

## Material and Methods

### Study area

The study was conducted at Muhimbili National Hospital (MNH), in Dar-es-Salaam, Tanzania. This is the largest and most densely populated region in Tanzania (population is 4.4 million; Population density of 3,133 persons per square km) [11]. The prevalence of malaria in children below five years in Dar-es-Salaam is 3.6% [12]. The birth prevalence of SCD in Tanzania is 6 per 1,000 live births, ranking Tanzania as the fourth country in the world (after Nigeria, Democratic Republic of Congo (DRC) and India) with the highest number of SCD births [13].

### Study population

The study included individuals seen at MNH between March 2004 and December 2013 within the framework of the Muhimbili Sickle Cell (MSC) programme which has been previously described [3,14]. Briefly, individuals are identified at paediatric SCD or haematology clinics or during hospitalization and are screened for SCD. Screening is done either due to clinical features suggestive of SCD, referral by a doctor or self-referral by individuals. Individuals found to have HbAA or HbAS receive counseling regarding their SCD status and are not required to return to the clinic. Some of these individuals may have more than one visit due to non-confirmation of SCD diagnosis at initial visit. Individuals with HbSS are enrolled into the MSC clinic, where detailed clinical and laboratory information are collected. Appointments for follow up visits are scheduled every 3–9 months. Care is provided to individuals with HbSS following national and hospital treatment guidelines. This includes folic acid, health education and malaria prevention using insecticide treated nets and prompt malaria case management. No chemoprophylaxis against malaria is provided as a preventive measure.

### Laboratory methods

SCD diagnosis (HbAA, HbAS and HbSS status) was done by alkaline hemoglobin electrophoresis (Helena, Sunderland, Tyne & Wear, UK). Confirmation of sickle phenotype and quantification of HbF was done by high performance liquid chromatography (HPLC) (Variant I analyzer, Bio-Rad, Hercules, CA, USA). For HbSS individuals younger than five years at enrolment into the MSC, HPLC was repeated after 5 years of age. This is because the level of HbF within an individual does not significantly change after the age of five [5]. Malaria screening was done at enrolment, during the clinic or at hospitalisation for patients with clinical features suggestive of malaria (fever or history of fever, general body pain, vomiting, and anaemia). Malaria diagnosis was done using rapid diagnostic test (RDT) (Parahit, Span Diagnostics; or Paracheck, Orchid Biomedical Systems) and/or blood slide (BS) using Giemsa stain.

### Ethical approval

The study was approved by the ethical committee of the Muhimbili University of Health and Allied Sciences (reference MU/RP/AEC/VOL XI/33). Written informed consent, in the local language (Kiswahili), was obtained from parents or guardians of children and from patients who were above 18-years old.

### Statistical methods

Information was checked for consistency before double entry into a database written in MySQLv5.0 (Sun Microsystems Inc, Santa Clara, California, USA). STATA 11 (Stata Corp, College Station, TX) and R 30.03 (http://www.R-project.org/) were used for analysis. The SCD status was categorized into three phenotypes: HbAA, HBAS and HbSS. Malaria infection was defined as any positive diagnosis determined by a positive blood slide using microscopy or by an RDT. This was irrespective of clinical features suggestive of malaria (fever or history of fever, general body pain, vomiting, and anaemia). The level of HbF was handled as a continuous variable. The MSC study is a cohort study and consists of longitudinal follow-up of individuals with HbSS. Therefore, HbSS have repeated clinic visits, and hospitalisations, with repeated measurements for malaria within individuals. The long period of follow-up and multiple visits of the MSC allowed evaluation of malaria, which has a low prevalence in HbSS and after the age of 5 years. Evaluation of the relationship between HbF, SCD status and malaria was done using the baseline visit as the date of HbF measurement as well as testing for malaria. Since the level of HbF is known to decrease in the first 5 years of life and the prevalence of malaria decreases with age, we also evaluated the association limiting analysis to measurements done in individuals above 5 years.

Continuous variables (age and HbF) variables were transformed toward normality using a square root transformation. Malaria prevalence was defined as the total number of positive tests over total number of tests. The proportions of malaria by SCD status were compared using a χ^2^-test or Fisher exact test if the expected count in one of the cells was less than five. HbF levels were compared using a t-test or Wilcoxon rank sum test in small samples. Logistic regression and Generalised Estimating Equation (GEE) models were used to assess the risks of malaria infection controlling for age and sex in single and repeated observations recorded during visits, respectively, to account for age dependent effects such as body immunity against malaria and HbF levels [5,6]. Models with and without interaction between SCD status and HbF fitted in logistic regression were compared using log likelihood ratio tests. A variable was considered statistically significant if its p-value was < 0.05.

## Results

**Fig 1:**
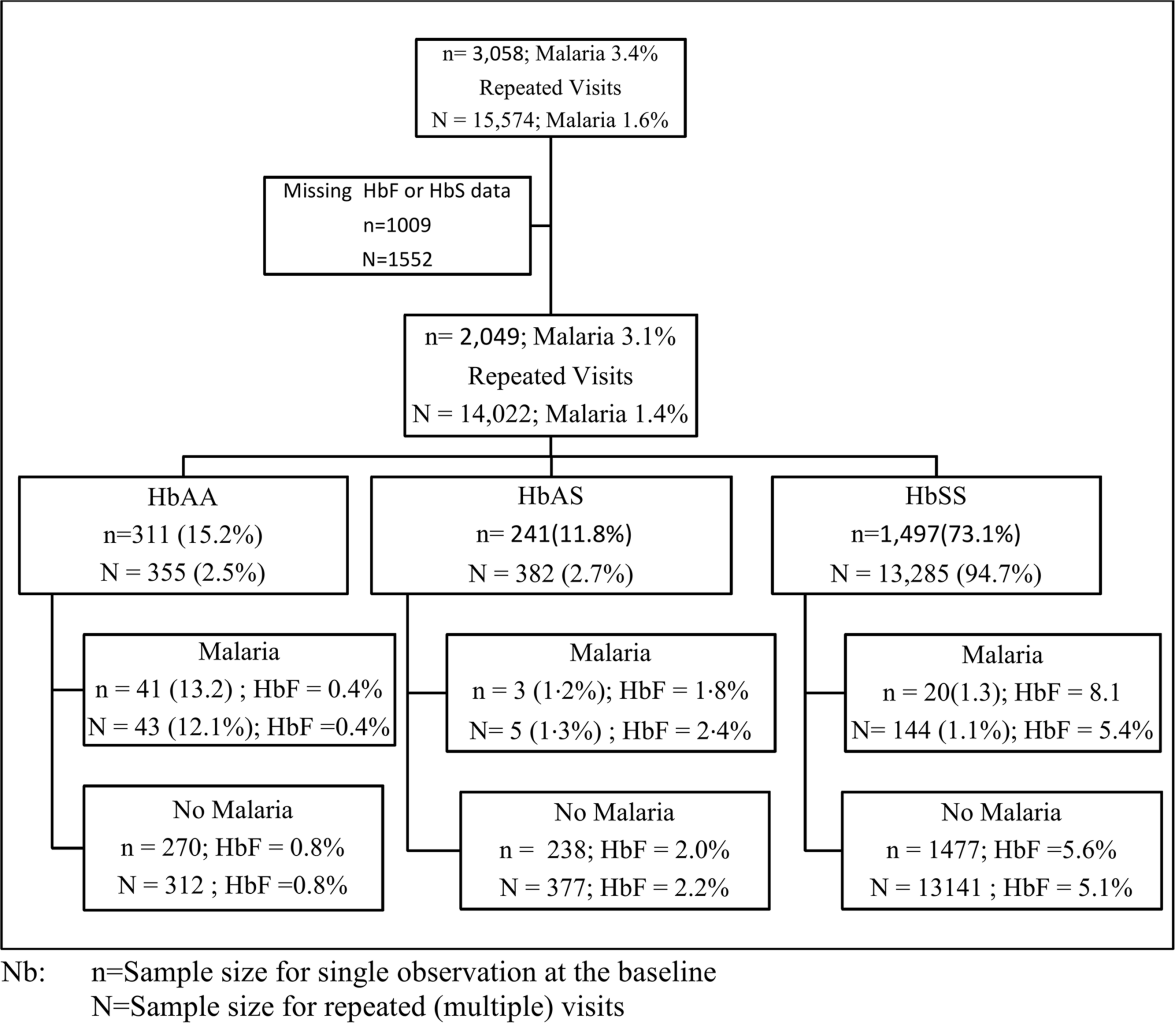
Participants in the malaria study in the Sickle Cell Disease programme at Muhimbili National Hospital.

2,049 individuals with age range 0–70 years, HbAA 311(15.2%), HbAS 241(11.8%) and HbSS 1,497(73.1%) with HbF and HbS data were screened for malaria (Fig 1).

General characteristics of the study population are presented in Table 1. The prevalence of malaria at baseline was 3.12%, with higher prevalence in children under-five years of age and decreasing prevalence with increasing age (χ^2^_*trend*_ = 9.8, p = 0.002). Individuals in the HbAA (13.18%) group had a significantly higher prevalence of malaria than HbAS (1.24%) or HbSS (1.34%) (χ_(2)_^2^ = 122.6, p<0.001).

**Table 1:** Characteristics of the study participants at baseline visit.

		Sample size	Malaria status		Test statistic
			Negative	Positive	(P-value)*
**Population size (%)**		2,049	1985 (96.9)	64(3·12)	
**Age group:**	**0–4y**	612	582 (95.1)	30 (4·9)	χ^2^_*trend*_ = 9·8 (0·002)
	**5–14y**	955	930 (97.38)	25 (2·62)	
	**15y+**	475	467 (98.32)	8 (1·68)	
	**Missing**	7	6 (85.71)	1(14·29)	
**Sex**	**Males**	1,032	997(96.61)	35(3·39)	χ^2^ = 0·493 (0·482)
	**Females**	1,017	988(97.15)	29 (2·85)	
**SCD status**	**HbAA**	311	270 (86.82)	41 (13·18)	χ^2^_(2)_ = 122·6 (<0·001)
	**HbAS**	241	238 (98.76)	3 (1·24)	
	**HbSS**	1,497	1477(98.66)	20(1·34)	
**HbF (mean, 95%CI)**					
	**Overall**	2,049	4.22 (4.02, 4.42)	1.89 (1.16, 2.79)	t = 4.84 (<0.001)
	**HbAA**	311	0.82 (0.65,1.01)	0.43 (0.27, 0.63)	t = 1.94 (0.053)
	**HbAS**	241	2.04 (1.64, 2.48)	1.79 (0.36, 10.75)	z = 0.021 (0.983)
	**HbSS**	1,497	5.59 (5.36,5.82)	8.10 (5.84, 10.71)	t = -2.250 (0.025)
**HbF (≥5years) (mean, 95%CI)**					
	**Overall**	1,430	3.74 (3.54, 3.94)	2.78 (1.39, 4.65)	t = 1.48 (0.138)
	**HbAA**	151	0.47 (0.33, 0.64)	0.18 (0.04, 0.41)	t = 1.48 (0.140)
	**HbAS**	127	1.13 (0.74, 1.60)	3.19 (0.13, 10.30)	z = -1.50 (0.135)
	**HbSS**	1152	4.74 (4.53, 4.96)	7.94 (5.29, 11.13)	t = -2.98 (0.003)

*All test statistics excludes missing values

There were 14,022 visits with information on malaria, HbF and SCD. The proportion of visits contributed by each SCD group was HbAA = 355 (2.5%), HbAS = 382 (2.7%) and HbSS = 13,285(94.7%). The prevalence of malaria for all visits was 1.36%, and when categorized by SCD status was HbAA 12.1%, HbAS 1.3% and HbSS 1.1%, (Fig 1). Analysis in individuals aged ≥5 as shown in S1 Fig and Table 1, shows that although malaria infection was lower in this age group, the results were consistent by SCD status as well as by HbF levels when compared to that of the population composed of all ages.

### Malaria in HbAA, HbAS and HbSS and level of HbF

**Fig 2:**
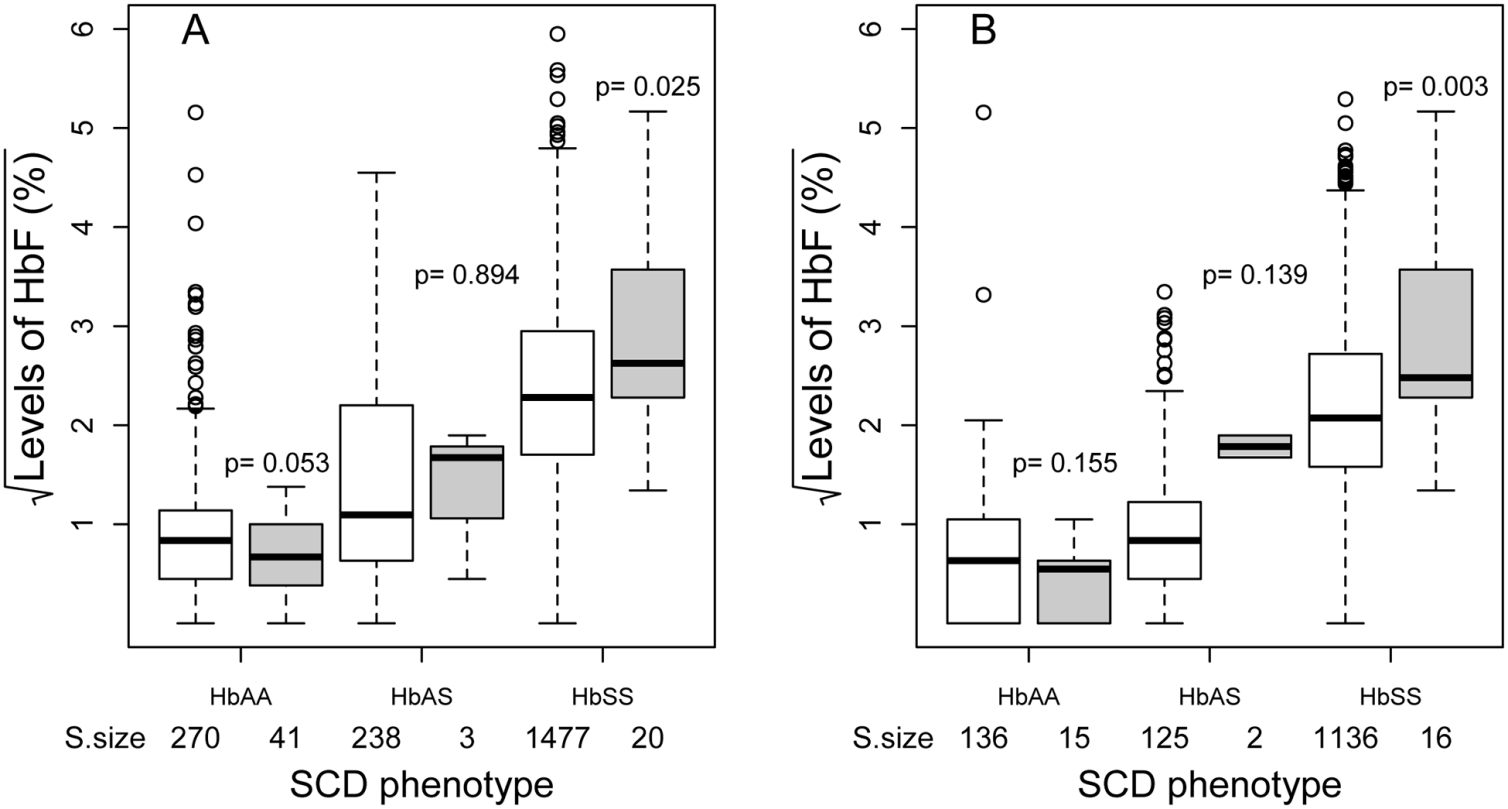
Association between malaria (Blank = Negative, Filled = Positive) by SCD and Foetal hemoglobin levels for individuals in all ages (A) and for ≥5 years (B). Sample size of each group is shown on the x-axis and p-values (p) in the graph were derived from t-tests (HbAA and HbSS) and Wilcox sign rank test (HbAS). The box show median (central line) and inter quartile range (IQR). The top whisker corresponds to the largest observation that is less than or equal to the 75th percentile + 1.5 x IQR. The bottom whisker corresponds to the smallest observation that is greater than or equal to the 25th percentile—1.5 x IQR, while open circles are the outliers.

Individuals with malaria had significantly lower HbF compared to those with no malaria 1.89% vs 4.22% (p<0.001). Mean HbF in individuals with malaria in the HbAA group was 0.43% (95%CI: 0.27, 0·63) compared to 0.82% (0.65, 1.01) in the no malaria group (p = 0.05). For the HbAS group, the mean HbF in the two groups were not significantly different (p = 0.983). However, for the HbSS group, the mean HbF was higher in the malaria group 8.10% (5.84, 10.71) compared to the no malaria group 5.59% (5.36, 5.82), p = 0.025, Table 1 and Fig 2A. When analysis was done for individuals aged ≥5 years, an age above which HbF is stable, a similar pattern of lower HbF in HbAA and high HbF in the HbAS and HbSS individuals with malaria infection was seen, Table 1, Fig 2B and S1 Table. However, the level of significance in HbAA was reduced due to small sample size of individuals with malaria.

Analysis of the effect of HbF and HbS on malaria was evaluated at baseline and during multiple visits (Table 2). A logistic model with an interaction term (SCD status and HbF) as presented in Table 2, was significant (Deviance = 9.64 with 2 degrees of freedom, p = 0.008) from the model with no interaction. Results from the interaction model indicate that in the HbAA group, an increase in HbF is associated with a decreased risk of malaria OR = 0.50 (95%CI: 0.28,0.90; p = 0.021), whereas for HbSS group, the risk of malaria increased with increase in HbF, OR = 2.94 (1.44, 5.98; p = 0.003).

**Table 2:** Coefficient estimates from multivariate logistic (single visit at the time of HbF measurement) and GEE (multiple visits) regression models of malaria in relation to SCD status, HbF levels and age for individuals of all ages.

		Single visit (n = 2,042)*		Multiple visits (n = 14,002)**		
Variable	OR	95% CI	P-value	OR	95% CI	P-value
**HbAA**	1			1		
**HbAS**	0.07	0.01, 0.52	0.009	0.07	0.02, 0.24	<0.001
**HbSS**	0.02	0.015, 0.09	<0.001	0.05	0.03, 0.10	<0.001
**HbF** ^**§**^	0.50	0.28, 0.90	0.021	0.52	0.34, 0.80	0.003
HbAS*HbF ^**§**^	1.59	0.44, 5.73	0.482	1.77	0.95, 3.32	0.073
HbSS*HbF ^**§**^	2.94	1.44, 5.98	0.003	2.01	1.27, 3.23	0.003
**Age** ^**§**^	0.80	0.63, 1.02	0.071	0.81	0.71, 0.91	0.001

^§^Transformed by square root

*Seven individuals with missing age excluded

**20 visits with missing age excluded

A similar pattern, with a stronger effect was seen when the model was fitted to data obtained from multiple visits, using GEE to take into account clustering within individuals (Table 2). Lower HbF levels were observed among individuals with malaria in the HbAA group OR = 0.52 (0.34, 0.80; p = 0.003) whereas in the HbSS group, higher HbF levels were observed among the individuals with malaria OR = 2.01 (1.27, 3.23; p = 0.003). Similarly results were obtained for individuals aged ≥5 years, with slightly higher odds ratios for the interaction between HbAS or HbSS and HbF levels, S1 Table.

## Discussion

Innate genetic immunity against malaria has been described in hemoglobin variants, particularly HbS and α-thalassemia. This has been the subject of considerable research, as understanding the mechanisms of protection that occur naturally in human could lead to development of interventions. However, the epistatic effect of various genetic variants and their resultant proteins offers additional insights into mechanisms of biological protection and geographical distribution on genes and disease. This study was designed to evaluate the effect of co-existence of HbF and HbS on malaria.

We initially set out to describe the independent association of HbS and HbF with protection against malaria. For HbS, we confirmed previous observations [1,2] that the prevalence of malaria in individuals with HbAS and HbSS was lower compared to those with HbAA. Less is known about the protective effect of HbF. Here we showed that a protective effect was evident in individuals with HbAA, with malaria being more common among those with lower levels of HbF. This effect had previously been described in newborns, in-vitro and studies in mice [4,15]. In an in-vitro study, Pasvol et al [4] showed that there were fewer parasites in cells containing HbF and there was a growth retardation of parasites in cells containing HbF. The distribution and growth of *Plasmodium falciparum* was compared in red blood-cells containing either adult or fetal haemoglobins. In in-vitro cultures, cord blood-cells were invaded more readily, but there was a paucity of parasites in cells containing HbF in the blood of infected infants aged 3–6 months. Some of studies have associated retardation of parasite growth in cell containing HbF with reduced ability of HbF-containing red blood cells to handle the oxidative stress imposed by developing parasites [16]. However, one of the recent studies associated HbF with impairment of binding of parasitized red blood cells to human microvascular endothelial cells, monocytes and nonparasitized erythrocytes [17].

This is one of the first epidemiological studies of this magnitude to suggest the protective effect of HbF against malaria. Our data suggest that there is negative epistasis between HbF and HbS on malaria protection. There was a loss of protection against malaria with increasing HbF levels in the HbSS group. Negative epistasis between HbS and α-thalassaemia has been described in Kenya [11] and another study has described epistatic variation between α-thalassemia and Haptoglobin (Hp) [18]. In the latter, individuals with Hp2-1 genotype in combination with heterozygote or homozygote α-thalassemia were protected from the disease, while those with Hp2-2 were not protected [18]. The high level of HF in individuals with malaria may also be a result of other factors such as stress erythropoiesis, drug induction and diseases [19,20]. Further studies on pathophysiology are required to elucidate the mechanism of high HbF in malaria. The occurrence of negative epistasis between HbS and HbF in malaria would have implications in our understanding of malaria protective mechanisms in areas with high prevalence of SCD and malaria. The interaction between these two haemoglobins and their effect on malaria may differ between populations with different levels of HbF, for example in Asia population where HbF levels are higher than African population [21,22]. Epistatic interactions between different malaria-protective haemoglobin variants may be one of the factors that determines their geographical distribution [22]

This study was undertaken within the epidemiological framework of a cohort study for HbSS and it had a limited sample size especially for HbAS and HbAA. Thus, this was not a random sample of the general population. This may have led to selection bias for those with malaria in the HbAA and HbAS groups but is unlikely to have biased selection of individuals with varying levels of HbF. Despite higher levels of HbF among individuals with HbAS infected with malaria; these were not significantly different from un-infected individuals. This may be the result of the small sample size and few malaria events in this population. Further studies should be done to evaluate these observations in population—based studies. Furthermore, additional research is needed to evaluate the effect of these haemoglobin variants on malaria parasite density as well as the quantitative effects between HbS and HbF on malaria. This can be done by exploring the effect of different levels of HbF and HbS on presence of malaria parasitaemia as well as the association with malaria parasite density. Further studies should examine the effect of HbF, HbS and the wider range of haemoglobin variants on malaria, clinical outcomes and immune responses to provide a more complete picture of the overall additive and epistatic contribution of genetic regulation in Africa. The interaction between HbS and HbF with other hemoglobin disorders like α-thalassemia as well as the mechanism of loss of protection against malaria infection with co-existence of high levels of HbF and sickle gene needs to be explored further. This study was not able to explore this relationship because of the low prevalence of malaria infection. Among 1,247 individuals with α- thalassemia data, none among the HbAA group and one in HbAS had malaria. Evidence suggests that although the risk of malaria is lower among individuals with SCD, this group has a higher risk of morbidity and mortality related to malaria. If indeed, the risk for malaria is much higher in those with higher levels of HbF, this will bring challenges in management of SCD in malaria-endemic areas. This is because interventions such as Hydroxyurea which increase HbF levels may inadvertently result in increasing the risk of malaria.

This is one of the first epidemiological studies to report on the epistatic interaction between HbF and HbS on malaria. This study opens up further questions that need to be explored to increase our understanding of the interactions of hemoglobin variants that occur in malaria endemic area. Such studies should involve population-based, epidemiological studies involving adequate numbers of individuals with HbAS, HbAA and HbSS in areas with high transmission of malaria. Further insight can be elucidated from in-vitro studies that explore the dynamics of malaria infection including the growth of malaria parasites under different levels of HbF and HbS.

## Supporting Information

S1 Fig Participants aged ≥5 years in the malaria study in the Sickle Cell Disease programme at Muhimbili National Hospital. (TIF)

S1 Table Coefficient estimates from multivariate logistic (single visit at the time of HbF measurement) and GEE (multiple visits) regression models of malaria in relation to SCD status, HbF levels and age for individuals aged ≥5 years. (PDF)
