# Supplementary figures and images for: Negative Epistasis between Sickle and Foetal Haemoglobin Suggests a Reduction in Protection against Malaria
Source: PLoS One. 2015 May 12;10(5):e0125929. doi: 10.1371/journal.pone.0125929 (PMC4428884; doi:10.1371/journal.pone.0125929)

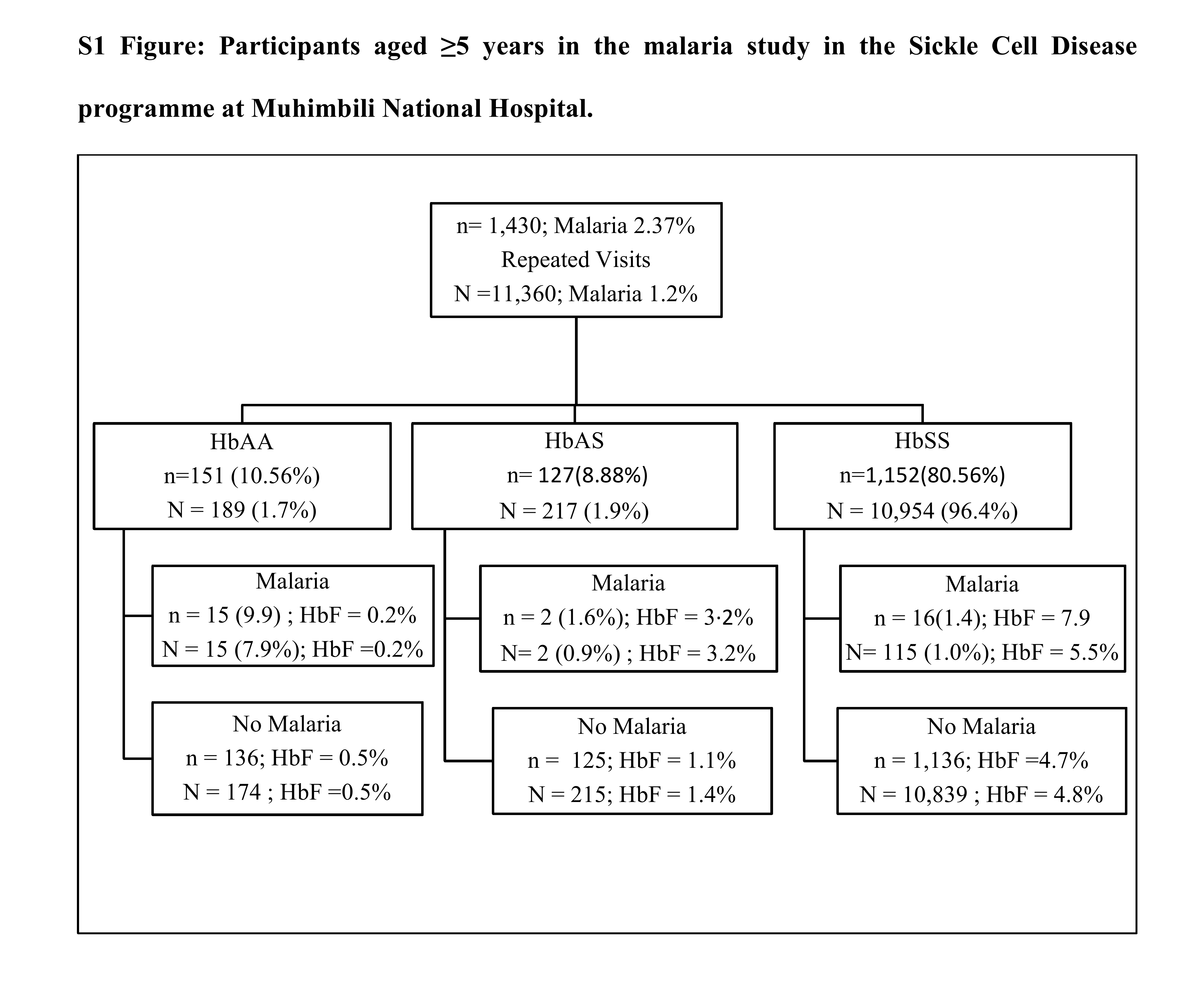

Supplement: S1 Fig — (TIF) [file pone.0125929.s001.tif]
