# Supplementary material for: Negative Epistasis between Sickle and Foetal Haemoglobin Suggests a Reduction in Protection against Malaria
Source: PLoS One. 2015 May 12;10(5):e0125929. doi: 10.1371/journal.pone.0125929 (PMC4428884; doi:10.1371/journal.pone.0125929)
Supplement: S1 Table — (PDF) [file pone.0125929.s002.pdf]

S1 Table: Coefficient estimates from multivariate logistic (single visit at the time of HbF measurement) and GEE (multiple visits) regression models of malaria in relation to SCD status, HbF level and age for individuals aged ≥5 years

	Single visit			Multiple visits		
**Variable**	**OR**	**95% CI**	**P-value**	**OR**	**95% CI**	**P-value**
**HbAA**	1			1		
**HbAS**	0.08	0.01, 0.55	0.011	0.05	0.01, 0.25	<0.001
**HbSS**	0.01	0.00, 0.07	<0.001	0.05	0.02, 0.13	<0.001
**HbF[§]**	0.36	0.11, 1.18	0.091	0.38	0.14, 1.04	0.061
**HbAS*HbF[§]**	3.32	0.86, 12.79	0.081	3.33	1.19, 9.31	0.022
**HbSS*HbF[§]**	5.53	1.54, 19.83	0.009	3.16	1.13, 8.9	0.029
**Age[§]**	0.59	0.38, 0.94	0.027	0.75	0.61, 0.92	0.005

[§]Transformed by square root
